# Survival Estimation Using Multistate Cormack–Jolly–Seber Models—The Case of the Bearded Vulture *Gypaetus barbatus* in Spain

**DOI:** 10.3390/ani14030403

**Published:** 2024-01-26

**Authors:** Inmaculada Navarro, Miguel Ángel Farfán, Juan Antonio Gil, Antonio Román Muñoz

**Affiliations:** 1 Departament of Animal Biology, Faculty of Science, Universidad de Málaga, Campus de Teatinos, 29071 Malaga, Spain; mafarfan@uma.es (M.Á.F.); roman@uma.es (A.R.M.); 2 Fundación para la Conservación del Quebrantahuesos, 50001 Zaragoza, Spain; jagil@quebrantahuesos.org

**Keywords:** vultures, ecology, population analysis, modelling, conservation, biodiversity

## Abstract

**Simple Summary:**

In the Aragonese Pyrenees, we studied the survival and productivity of bearded vultures, an endangered species known for its diet of bones. Using data from 1987 to 2020, we found that the survival rates for juveniles, subadults and adults were 90%, 95% and 92%, respectively. Overall, the species’ survival has improved, but juvenile survival is a concern. Productivity in the area is decreasing. Our research helps identify vulnerable age groups, guiding conservation efforts. The study suggests being cautious about feeding points to protect the species.

**Abstract:**

The bearded vulture (*Gypaetus barbatus*) is an endangered species with a specialist osteophagous (bone) diet. We estimated the survival and productivity of this vulture in the Aragonese Pyrenees, where the main population of the species in Europe is found. We used a database covering a period of 33 years (1987–2020). To estimate the probability of survival, we used Cormack–Jolly–Seber models with a Bayesian approach. Our models estimated a survival rate of 0.90 ± 0.08 in juveniles, 0.95 ± 0.04 in subadults and 0.92 ± 0.05 in adults. The survival probability increased over the study period in adults and subadults but not in juveniles. By contrast, productivity decreased over the same period. Our study provides updated information on the status of two demographic parameters of great importance to the species and allows us to identify the most vulnerable age classes and to plan conservation actions to improve the situation of the species in a territory that is a donor of specimens for reintroduction projects. The estimated survival values suggest that more caution should be exercised when planning these feeding points according to the use the species makes of them.

## 1. Introduction

Continuous monitoring of endangered species is essential for conservation [1]. Biodiversity monitoring can be challenging, leading to errors due to environmental factors, imperfect detection [2] and other factors, such as insufficient information, that can lead to errors [3]. Continuous monitoring provides valuable information for understanding changes in ecosystems, identifying emerging threats and designing effective management and conservation strategies [1]. This is why long-term population monitoring programs are essential for accurate knowledge on wildlife populations [3]. The monitoring of species and the use of statistical models to deal with imperfect detection allow us to properly understand the population dynamics, better understand their ecology and manage them efficiently [4].

In wild populations, survival is one of the demographic parameters governing population dynamics [5]. To estimate the probability of survival of birds, capture–recapture models are frequently used, with the Cormack–Jolly–Seber (CJS) model [6,7,8] being the most commonly used methodology [5]. The CJS model ascribes probabilities to every conceivable capture history using two sets of parameters. These represent the capture probabilities, signifying the chances that animals present during a single capture event are indeed captured, and the survival probabilities, indicating the likelihood that animals alive on one capture occasion remain alive by the next. While these probabilities are permitted to vary over time, the CJS model enforces the assumption that the capture and survival probabilities remain consistent for all animals in the population during a single capture event. Additional assumptions within the model include the consideration that capture events are instantaneous occurrences, no harm befalls the animals during capture, emigration is permanent and individuals act autonomously without influence from one another [9]. Following this, statisticians and ecologists have introduced extensions to the CJS model, permitting the adjustment of capture and survival rates based on covariates. Pollock (2002) [10] offers an extensive overview of these techniques, which include the adoption of multistate models and the application of generalised linear models (GLMs) [9]. In a multistate model, the capture and survival rates can independently fluctuate among animals, each of which belongs to a finite number of states defined by one or more factors. A significant benefit of employing a multistate model lies in its capacity to integrate covariates that are not only time-varying but also specific to each individual. This is achieved by utilising a Markov chain to depict individuals’ transitions between states and to factor unobserved covariate values into the model likelihood [4,9].

The bearded vulture, with a specialised osteophagous diet [11], inhabits mountain ranges characterised by a steep topography and rocky nesting sites. This bird also frequents adjacent plains and plateau areas, where it relies on extensive open spaces with minimal vegetation to locate food [12,13]. While it depends on thermal currents and wind for gliding, this reliance is notably less than that of most other vultures [12,13].

Bearded vultures have a distribution across mountainous regions spanning Eurasia and Africa [14]. Historically, they occupied all major mountain systems within the Iberian Peninsula [15,16]. Presently, the Spanish population is confined to a breeding population in the Pyrenees, specifically Catalonia, Aragon and Navarre. In 2019, this population consisted of 186 reproductive units (RU: each RU is defined as a breeding pair or, if applicable, polyandrous trios), accounting for over 80% of the European population [11]. Additionally, two more nuclei have emerged naturally, with one RU located in the Vasco–Navarras mountain chains (Guipúzcoa) and another in the Moncayo Massif (Zaragoza). Furthermore, through reintroduction initiatives, two new nuclei have been established: one in the Cantabrian Mountains (Asturias) and the other in the Cazorla mountain chain (Jaén) [11].

The bearded vulture’s habitat is confined to mountain regions characterised by extensive livestock farming [11]. Livestock farming plays a vital role in the conservation of this species, particularly in the Pyrenees, where the bearded vulture’s diet primarily consists of domestic ungulates (*Ovis* and *Capra*) and wild ungulates (*Rupicapra*) [14,17]. Extensive livestock farming represents a pivotal activity in developed countries’ mountain ecosystems [18,19]. In the Aragonese Pyrenees, it holds essential significance [20], leading to the maintenance of extensive meadows in the region, subject to regular grazing on both public and private lands. This practice carries significant economic, social and ecological value [21].

The bearded vulture is a large and long-lived species, with a clutch size of two eggs, of which only one chick survives due to inter-sibling competition [22]. Although it is a monogamous species, polyandrous trios are easily observed in the Pyrenees, probably due to the density dependence caused by the increase in the population and the decrease in optimal territories for the species [22]. The estimated age of first breeding is 10.31 years for the Pyrenean population [22].

The bearded vulture is considered at the European level to be one of the most endangered birds of prey (Annex I, EU Birds Directive 79/409/EEC and 2009/147/EEC, Appendix II of the Bern Convention, Bon Convention and CITES). Within North Africa, it attains the classification of Critically Endangered based on the IUCN criteria, owing to its exceedingly rare occurrence and small population [23]. Likewise, in South Africa, where the subspecies G. b. meridionalis is found, it is also classified as Critically Endangered [24]. As of 2019, its conservation status in Europe, as per the IUCN, is deemed Vulnerable. In Spain, it holds the designation of Endangered according to the Spanish Catalogue of Threatened Species, under RDL 139/4 2011 February.

The main objective of this study is to define the status of the bearded vulture population in the Aragonese Pyrenees. The management decisions on a donor population, like the Aragonese Pyrenees population, must take into account possible fluctuations in the demographic and reproductive parameters, making a continuous and updated analysis of these parameters necessary [25].

## 2. Materials and Methods

- Study area

**Figure 1:**
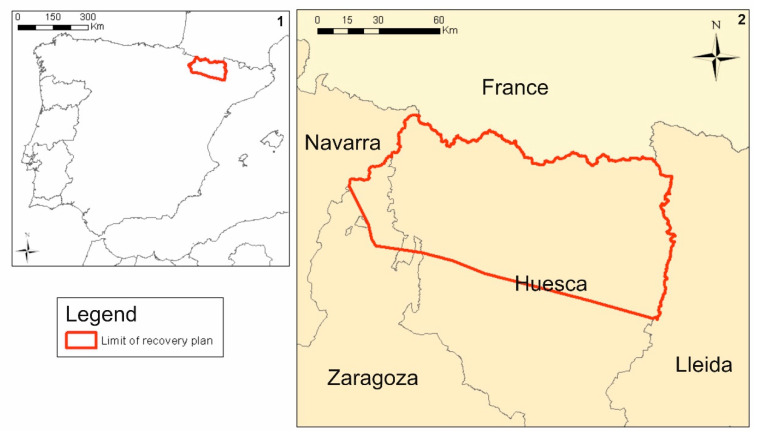
Study area. 1: Iberian Peninsula context; 2: Pyrenean context.

The geographical context of this study was the Aragonese Pyrenees (northern Spain), where the main population of the species in Spain is located [11] (Figure 1). The study area is delimited by the recovery plan for the species: it covers 9537 km^2^ and fully includes the Ordesa y Monte Perdido National Park, declared a World Heritage Site by UNESCO. The study area thus encompasses part of the bearded vulture population present in the Pyrenees.

The Aragonese Pyrenees are made up of a large number of rocky cliffs and valleys with livestock activity [26]. It is in this type of ecosystem that the bearded vulture finds its optimal habitat [11].

- Data collection

CMR (Capture–Mark–Recapture) data have been collected over a long-term study. For this purpose, 227 birds of known age were marked over a period of 33 years (1987–2020). The capture and tagging of juveniles was carried out at nests, and subsequent ring-reading was done at active nests and SFS. The marking process consisted of ringing and attaching wing bands that can be seen from a distance. The wing bands are alphanumerically coded and coloured differently for each individual so that each bird can be easily identified. Sightings were carried out by monitoring breeding territories, as well as by visiting weekly SFS (supplementary feeding stations) located in the study area (Figure 1). Sightings occurred during winter counts. From these sightings, a database of the absence and presence of sightings of each bearded vulture has been constructed for every year.

For the analysis of trends in the reproductive parameters and in the number of pairs, data derived from the monitoring program of the species from 1990 to 2021 have been used.

- Survival analyses

The estimation of the probability of survival of the bearded vultures was carried out by means of a multistate Cormack–Jolly–Seber (CJS) model [2]. The CJS model used is an age-dependent model, which can be formulated as follows:

logit (ϕi,t) = βx(i,t) + εi

εi~Normal (0, σ2),

where βx(i,t) demonstrates the effects of the age class x of individual i at time t, and εi is the year random effect.

For a survival analysis grouped by age class, we defined three classes according to plumage characteristics [22]. We have defined juvenile birds as those under 2 years of age, subadults as birds between 2 and 6 years of age and adults as those over 6 years of age. In total, the database consisted of 227 birds of known age.

The statistical analyses were performed in R version 4.3.1 [27] and the JAGS version 4.3.1 [28] for programming Markov Chain Monte Carlo (MCMC) methods for Bayesian models and thus assessing the convergence of the random samples [29]. The “jagsUI” R package [30] was used to call JAGS from the R software. We specified 150,000 iterations, a burn-in of 5000 and a rate of thin of 3 and 3 chains. We used Brooks–Rubin–Gelman diagnostics [31]. The results were always less than 1.1, indicating that the chains converged to a stable distribution [2].

- Productivity trend analyses

An analysis of bearded vultures’ productivity from 1995 to 2020 was carried out. Productivity as a reproductive parameter of the bearded vultures was defined as follows: total number of chicks fledged/total number of pairs [11].

To examine the relationship between the independent variable (year) and the dependent variable (productivity), Spearman’s coefficient [32] was used.

- Number of pairs trend analyses

To understand the productivity results, an analysis of the evolution of the number of pairs in the study area was carried out. Pairs could be composed of two or three individuals, forming polyandrous groups in the latter case. The database covers 33 years, from 1990 to 2021.

To explore the connection between the independent variable (year) and the dependent variable (number of pairs), we used Spearman’s coefficient [32].

## 3. Results

**Figure 2:**
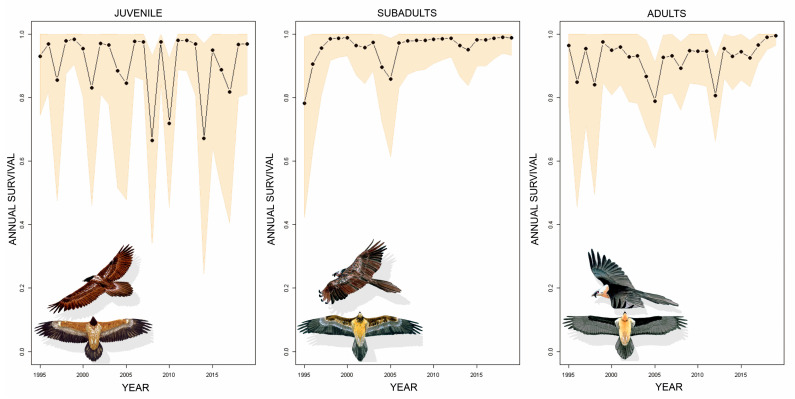
Interannual variation in mean annual survival for the three age classes of bearded vultures in the Aragonese Pyrenees from 1995 to 2019. Survival means are shown as dots and lines, and the 95% confidence interval around the means is shaded. Bearded vulture illustrations: Xabier Parellada.

Juvenile survival was estimated at 0.907 (0.821–0.993), subadult survival at 0.958 (0.915–1) and adult survival at 0.924 (0.924–0.979). Regarding the trends in this parameter in the Aragonese Pyrenees, we observed a weak positive trend in subadults and adults in recent years, while there was no trend in juveniles, with large interannual variations, especially in recent years (Figure 2).

The resighting rate was 0.88 (0.871–0.889) for the whole set of individuals. For juvenile individuals, the resighting rate was 0.881 (0.871–0.889); for subadult individuals, the resighting rate was 0.88 (0.871–0.889); while adult individuals had the lowest resighting rate at 0.879 (0.87–0.889).

**Figure 3:**
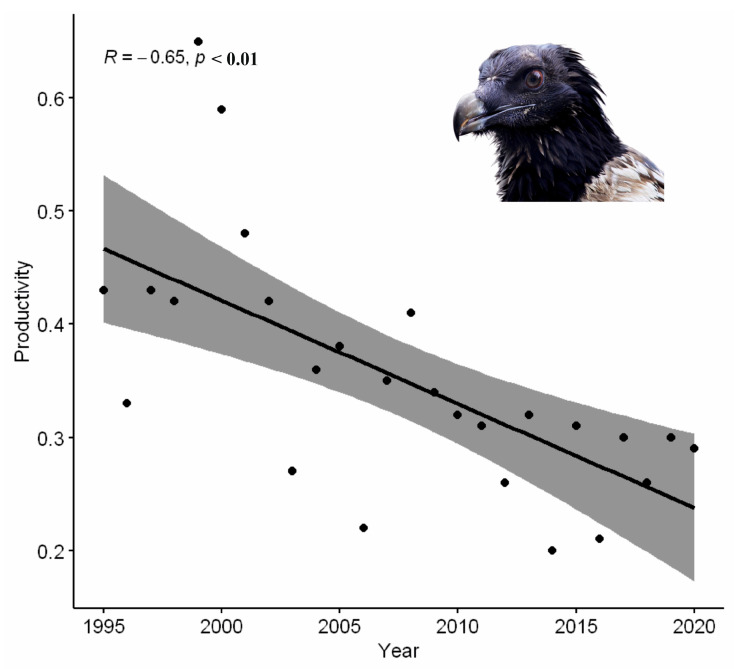
Interannual variation in productivity of bearded vultures in the Aragonese Pyrenees from 1995 to 2020.

Interannual variability was observed in their productivity (Figure 3). Spearman’s correlation between productivity and the study time period was negative and significant at a 99% confidence level (R = −0.65, *p* < 0.01).

**Figure 4:**
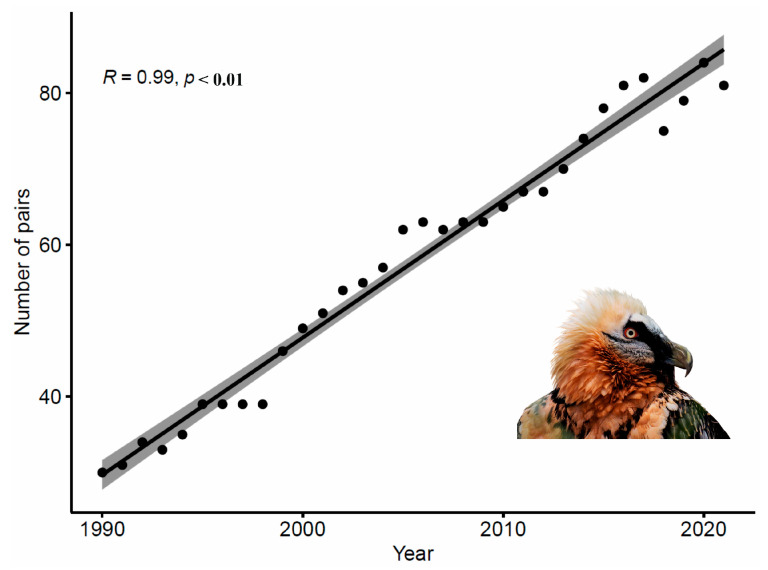
Interannual variation in number of pairs of bearded vultures in the Aragonese Pyrenees from 1990 to 2020.

Likewise, interannual variability was observed in the number of pairs (Figure 4). Spearman’s correlation between the number of pairs and the study time period was positive and significant at a 99% confidence level (R = 0.99, *p* < 0.01).

## 4. Discussion

Investigating the survival dynamics of the bearded vulture in the Pyrenees is pivotal to biodiversity conservation efforts. Our findings reveal interannual variations in survival across all age classes, with a slight positive trend in the Aragonese Pyrenees in recent years, except for the interannual variation in juvenile survival, particularly recently. Monitoring this variation is crucial, given the vulnerability of this species to extinction, as emphasised by Sergio et al. [33]. The lower survival among juveniles may be linked to intraspecific competition for food, indicating potential density-dependent effects. Understanding these nuances is imperative for effective conservation strategies not only in the Pyrenees but in the Iberian Peninsula as a whole.

The negative trend in productivity and the increase in the number of pairs could also reflect a density-dependent population status, as other studies have already pointed out for the Pyrenees as a whole [11,22]. These data, together with the low stability of juvenile survival over time, indicate that the species is fragile in the long term and that its continuity is not assured, as it is vulnerable to stochastic processes. In addition to the density-dependence phenomena, feeding, not so much in terms of quantity as in terms of quality, is important and should be assessed as a modulating factor in juvenile survival. Wild ungulates with traces of lead from hunting and other toxins found in carcasses can negatively influence reproductive parameters and the survival of the species [22].

The sharp decline in survival in all age classes in 2005 could be due to the health regulations in Spain from 2005 to 2011 due to Bovine Spongiform Encephalopathy, as trophic resources decreased. Perhaps individuals had to move away from the study area due to these food restrictions. This decline in the estimated survival during the health restrictions highlights the need to find a balance between public health protection and biodiversity conservation.

The intriguing disparity in survival rates between subadult and adult bearded vultures presents a matter that warrants careful consideration. The observed higher subadult survival values compared to those in adults raise questions about the underlying factors influencing these patterns, presenting two possible scenarios: first, there may be a potential underestimation of adult survival using the models, possibly attributed to the higher re-sighting rate in subadults. Alternatively, the elevated survival rates in subadults might be indicative of an increased trophic availability, as they are more likely to frequent supplementary feeding sites (SFS) compared to territorial adults [22]. The SFS have undeniably exerted a pivotal role in the proliferation of bearded vulture populations. However, it is imperative to acknowledge that their impact may be multifaceted, encompassing not only positive outcomes but also noteworthy negative implications. It should be noted that the convenience of sightings at these SFS exceeds that of other, often isolated or remote, locations. Sightings of ringed or wing-tagged individuals are much easier at these locations and, consequently, a substantial proportion of sightings tend to occur at these frequented sites, which attract mainly non-breeding individuals [22]. Moreover, considering the outcomes of the productivity trend analysis under a density-dependent scenario, coupled with the pronounced philopatry observed in Pyrenean specimens, there is a pressing need for enhanced management of the SFS. One potential strategy could involve reducing the predictability of food inputs, thereby encouraging specimens to disperse [34].

Even in a territory marked by a high density of bearded vultures, there are some disconcerting features influencing the future of the species (exposure to toxins, environmental issues, etc.), a concern echoed not only in the Aragonese Pyrenees but throughout the Pyrenean region, as highlighted by Margalida et al. [22]. In light of our findings, we propose a reflective reassessment of the supplementary feeding stations strategy in the Aragonese Pyrenees. This aims to address the identified issues and design specific studies to further explore the potential relationship between supplementary feeding stations (SFS) and toxins in the dynamics of the species. Furthermore, it is imperative to continue monitoring for the implementation of effective and science-based management practices. The ongoing tracking of key parameters ensures a comprehensive understanding of the evolving dynamics and facilitates adaptive management strategies in the context of bearded vulture conservation in the Aragonese Pyrenees.

## 5. Conclusions

Our investigation into the survival dynamics of the bearded vulture in the Aragonese Pyrenees highlights the critical role of understanding these dynamics in biodiversity conservation. While we observed a positive overall trend in the Aragonese Pyrenees, the concerning decline in juvenile survival, potentially linked to food competition and density-dependent effects, emphasises the species’ vulnerability. The negative trend in productivity, influenced by factors like lead exposure and toxins in prey, raises alarms about the species’ long-term fragility. A complex interplay of factors, including the impact of health regulations and the disparity in survival rates between subadults and adults, underscores the need for careful management of supplementary feeding stations to balance positive outcomes with potential drawbacks. Our findings call for a reflective reassessment of conservation strategies, particularly in the Aragonese Pyrenees, and emphasise the importance of ongoing monitoring and adaptive management practices to ensure the survival of the bearded vulture in this region.

## Data Availability

The data on bearded vulture counts in the Aragonese Pyrenees belong to the Government of Aragon and can be accessed on formal request. The data presented in this study are available in this article and from the corresponding author upon reasonable request.

## References

[1] Robinson N.M., Scheele B.C., Legge S., Southwell D.M., Carter O., Lintermans M., Lindenmayer D.B. (2018). How to ensure threatened species monitoring leads to threatened species conservation. Ecol. Manag. Restor..

[2] Kéry M., Schaub M. (2012). Estimation of survival from Capture-Recapture Data Using the Cormack-Jolly-Seber Model. Bayesian Population Analysis Using WinBUGS. A Hierarchical Perspective.

[3] Likens G., Lindenmayer D. (2018). Effective Ecological Monitoring.

[4] Jiménez J. (2017). Modelos Jerárquicos Bayesianos Aplicados al Seguimiento de Fauna. Ph.D. Dissertation.

[5] Jankowiak L., Wysocki D., Greño J. (2016). Survival and Site Fidelity of Urban Blackbirds *Turdus merula*—Comparison of Cormack-Jolly-Seber and Barker Models. Acta Ornithol..

[6] Cormack R.M. (1964). Estimates of survival from the sighting of marked animals. Biometrika.

[7] Jolly G.M. (1965). Explicit estimates from capture-recapture data with both death and immigration-stochastic model. Biometrika.

[8] Seber G.A. (1965). A Note on the Multiple-Recapture Census. Biometrika.

[9] Bonner S., Schwarz C. (2006). An extension of the Cormack-Jolly-Seber model for continuous covariates with application to *Microtus pennsylvanicus*. Biometrics.

[10] Pollock K.H. (2002). The use of auxiliary variables in capture-recapture modelling: An overview. J. Appl. Stat..

[11] Margalida A., Martínez J. (2020). El Quebrantahuesos en España: Población Reproductora en 2018 y Método de Censo.

[12] Ferguson-Lees J., Christie D.A. (2001). Raptors of the World.

[13] Orta J., De Juana E., Marks J.S., Sharpe C.J., García E.F. (2020). Bearded vulture (*Gypaetus barbatus*). Birds of the World.

[14] Gil J.A., Báguena G., Díez O. (2019). El Quebrantahuesos y sus Montañas: Biología y Conservación.

[15] Hiraldo F., Delibes M., Calderón J. (1979). El quebrantahuesos (*Gypaetus barbatus*) (L.). Sistemática, Taxonomía, Biología, Distribución y Protección.

[16] Gil J.A. (2020). Pasado, Presente y Futuro del Quebrantahuesos (Gypaetus barbatus) en el Maestrazgo (Teruel).

[17] Lozano P.J., Jauregui M. (2021). Utilización de los sistemas de información geográfica para la modelización de la conectividad biogeográfica: El ejemplo del quebrantahuesos (*Gypaetus barbatus*) en el Pirineo Occidental. Lurralde Investig. Espac..

[18] Pătru-Stupariu I., Hossu C.A., Grădinaru S.R., Nita A., Stupariu M.S., Huzui-Stoiculescu A., Gavrilidis A.A. (2020). A Review of Changes in Mountain Land Use and Ecosystem Services: From Theory to Practice. Land.

[19] Muñoz E., Martín D., Tenza A., Casasús I., Bernués A., Villalba D. (2021). Exploración preliminar del impacto del cambio climático sobre los sistemas ganaderos de montaña en el Pirineo aragonés. Proceedings of the XIX Jornadas Sobre Producción Animal.

[20] Estévez-Moreno L.X., Miranda-de la Lama G.C., Villarroel M., García L., Abecia J.A., Santolaria P., María G.A. (2021). Revisiting cattle temperament in beef cow-calf systems: Insights from farmers’ perceptions about an autochthonous breed. Animals.

[21] Reiné R.J. (2017). ¿Por qué investigar los pastos del Pirineo aragonés?. Lucas Mallada Rev. Cienc..

[22] Margalida A., Jiménez J., Martínez J.M., Sesé J.A., García-Ferré D., Llamas A., Razin M., Colomer M., Arroyo B. (2020). An assessment of population size and demographic drivers of the Bearded vulture using integrated population models. Ecol. Monogr..

[23] Allaoui I., Cherkaoui S. (2018). New breeding record of Lammergeier (*Gypaetus barbatus barbatus*) in Morocco and proposals for its conservation. Go-South Bull..

[24] Krüger S.C., Taylor M.R., Peacock F., Wanless R.W. (2015). Bearded vulture *Gypaetus barbatus*. The Eskom Red Data Book of Birds of South Africa, Lesotho and Swaziland.

[25] Anders A., Dearborn D., Faaborg J., Thompson F. (1997). Juvenile Survival in a Population of Neotropical Migrant Birds. Conserv. Biol..

[26] García-Ruiz J.M., Valero-Garcés B.L. (1998). Historical geomorphic processes and human activities in the Central Spanish Pyrenees. Mt. Res. Dev..

[27] R Core Team (2021). R: A Language and Environment for Statistical Computing.

[28] Plummer M. (2015). JAGS Version 4.0 User Manual; Lyon, France. http://sourceforge.net/projects/mcmc-jags/.

[29] Du H., Ke Z., Jiang G., Huang S. (2022). The Performances of Gelman-Rubin and Geweke’s Convergence Diagnostics of Monte Carlo Markov Chains in Bayesian Analysis. J. Behav. Data Sci..

[30] Kellner K., Meredith M., Kellner M.K. (2019). Package ‘jagsUI’. A Wrapper Around ‘rjags’ to Streamline ‘JAGS’ Analyses.

[31] Brooks S., Gelman A. (1998). General methods for monitoring convergence of iterative simulations. J. Comput. Graph. Stat..

[32] Spearman C. (1904). The Proof and Measurement of Association Between Two Things. Am. J. Psychol..

[33] Sergio F., Tavecchia G., Blas J., Tanferna A., Hiraldo F. (2021). Demographic modeling to fine-tune conservation targets: Importance of pre-adults for the decline of an endangered raptor. Ecol. Appl..

[34] Margalida A., Pérez-García J.M., Afonso I., Moreno-Opo R. (2016). Spatial and temporal movements in Pyrenean bearded vultures (*Gypaetus barbatus*): Integrating movement ecology into conservation practice. Sci. Rep..

